# Cloning, Expression and Functional Characterization of *V. vinifera* CAT2 Arginine Transporter

**DOI:** 10.3390/ijms26136259

**Published:** 2025-06-28

**Authors:** Lorena Pochini, Teresa Maria Rosaria Regina, Maria Iolanda Cerbelli, Nicoletta Gallo, Federica Costantino, Michele Galluccio, Cesare Indiveri

**Affiliations:** 1 Laboratory of Biochemistry, Molecular Biotechnology and Molecular Biology, Department of Biology, Ecology and Earth Sciences (DiBEST), University of Calabria, Via P. Bucci 4c, 6c, 87036 Arcavacata di Rende, Cosenza, Italy; lorena.pochini@unical.it (L.P.); teresa.regina@unical.it (T.M.R.R.); ioly00cerbelli@gmail.com (M.I.C.); nico.gallo1996@libero.it (N.G.); fedecostantino91@gmail.com (F.C.); cesare.indiveri@unical.it (C.I.); 2 Institute of Biomembranes, Bioenergetics and Molecular Biotechnology (IBIOM), National Research Council of Italy (CNR), Via G. Amendola 122/O, 70126 Bari, Italy

**Keywords:** plant nitrogen, amino acid, transporter, secondary metabolites

## Abstract

The amino acid membrane transporters of grape species take part in metabolic pathways that play crucial roles in nitrogen trafficking and in the synthesis of secondary metabolites. Therefore, identifying these amino acid transporters and defining their functional properties might have further applications in crop improvement and, hence, relevance to human nutrition. The *VvCAT2* (Cation Amino acid Transporter) transporter cDNA has been isolated and cloned into a specific plasmid for over-expression in *Escherichia coli*. The expressed protein, after purification by Ni^2+^-chelating chromatography, has been functionally characterized in an experimental model of proteoliposomes by measuring the uptake of radiolabeled compounds. Arginine was revealed to be the best substrate, confirming the role of CAT2 in nitrogen trafficking in plant cells and within sub-cellular spaces, given its plausible localization in vacuoles. The transporter activity is modulated by pH, osmotic imbalance and ATP. The transport kinetics have been measured. Overall, the obtained data indicate the capacity of *Vv*CAT2 in transporting arginine, making it a possible target for crop improvement with a relevance to human health.

## 1. Introduction

The grapevine, *Vitis vinifera,* is one of the most important grape species in cultivation worldwide [1]. Its importance is related to the use of table grapes, to the production of wine and juice, and to the extraction of antioxidant compounds that are relevant to human therapy [2,3,4]. Therefore, the study of processes that may be related to or contribute to plant growth, development, reproduction and tolerance to biotic or abiotic stress is essential for the possible improvement of crops and for the extraction of secondary metabolites. Among the fundamental processes for plant growth, there is the shuttling of the nitrogen that is involved in both plant development and cell signaling. Membrane transporters for amino acids are required for either long-distance or intracellular nitrogen shuttling [5]. In this context, the vacuole plays a central role in fruit growth and quality. This organelle was originally considered responsible for cell turgor; subsequently, it was shown that the vacuole is also involved in cell growth, pH homeostasis, stress responses [6,7,8], signal transduction [7,8,9], digestion of proteins and storage of many compounds, such as ions, metabolites and water. Vacuoles can also accumulate toxic compounds [6,10]. Many proteins cooperate in performing the vacuole functions [6,7,10], including the transporters that are located in the vacuolar membrane with specificity towards nutrients and metabolites. In particular, transporters for amino acids with a high nitrogen content, such as arginine, are crucial for nitrogen exchange [6,10,11]. Thus, identifying and characterizing amino acid vacuole transporters is the focus of many research studies in multiple plant species [12]. Indeed, vacuolar nitrogen pools may be a target for increasing the yield of secondary metabolites. Despite the progress in genome sequencing and annotation in various organisms, many genes encoding transport proteins are still predicted and have not been definitively identified or characterized [12]. Many genes that encode amino acid transporters have been identified in plants [13]. These transporters are classified into two superfamilies: the amino acid-polyamine/choline (APC) and the amino acid transporter superfamily (ATF) [14]. Among the vacuolar transporters, there are the Cation Amino acid Transporters (CATs), members of the APC transporter family [5,13,14,15,16,17,18,19,20,21], one of which has been identified and characterized in *Solanum lycopersicum* by our group [22]. Other *CAT* genes have been identified in other species [16,20,23,24]. However, the molecular mechanisms of amino acid transport mediated by the encoded proteins across the vacuolar membrane remain poorly described due to the inaccessibility of the tonoplast from the extracellular environment [13,25,26,27]. Among the few exceptions, the *Cs*CAT2, a tonoplast-localized transporter in *Camellia sinensis,* has been cloned into a pYES2 vector for transformation into a yeast mutant strain. In this system, an H^+^-dependent theanine transport mechanism was shown [18]. *Cs*CAT2 expression in the roots of tea plants was significantly induced by theanine feeding and was also induced by cold stress [24]. Also, the vacuolar CAT2 from *S. lycopersicum* has been identified and characterized by exploiting a different experimental model: the proteoliposome system harboring the protein produced by *E. coli* expression [22]. In this system, we revealed the competence of *Sl*CAT2 for ATP-regulated arginine transport [22]. Despite the importance of *V. vinifera* in human health, little information is available on vacuolar transporters or transporters of other organelles in this plant species [28]. A tonoplast proteomic analysis [1] indicated the existence of at least 161 transport proteins. Among these, the *CAT2* gene from *V. vinifera*, as well as its protein product, has never been described or analyzed so far. Nevertheless, this transporter should be involved in nitrogen shuttling in vacuoles, thus being a target for improving plant growth, nutritional quality and yields of secondary metabolites. Therefore, the mRNA transcript encoding the CAT2 protein was isolated from leaves and cloned into an appropriate expression vector for protein production in bacteria and subsequent characterization in the proteoliposome experimental model that allows for information to be obtained on the function and regulation of transporters located in subcellular membranes in the absence of interference by other transporters.

## 2. Results

### 2.1. Cloning, Expression and Purification

The total RNA extracted from *V. vinifera* berry flowers was reverse transcribed, and the cDNA obtained from this reaction was used as a template to amplify the *VvCAT2* cDNA by RT-PCR. Following cloning in the pET-21a(+) vector, the sequence of the clone was verified by Sanger sequencing (Supplementary Figure S1). The plasmid harboring *VvCAT2* was transformed into an *E. coli* Rosetta(DE3)pLysS strain that supplies tRNAs that are specific for eukaryotic codons [29], and has already been successfully used for the expression of *Sl*CAT2 transporter [22].

**Figure 1:**
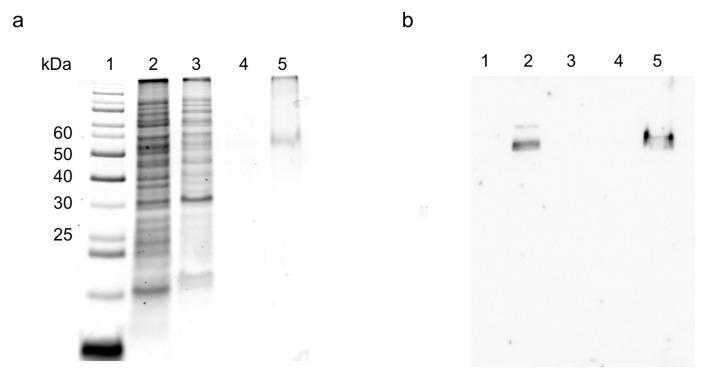
Purification of *Vv*CAT2 transporter. (**a**) Proteins were separated by SDS-PAGE: lane 1, page ruler prestained plus marker; lane 2: insoluble fraction of induced cell lysate solubilized before IMAC loading; lane 3: passthrough fraction containing unbound proteins; lane 4: fraction of proteins eluted with washing buffer with 10 mM imidazole added; and lane 5: fraction of protein eluted with 50 mM imidazole. (**b**) Western blotting of sample loaded as in (**a**).

For the production of the *Vv*CAT2 protein, the protocol previously adopted for the *Sl*CAT2 ortholog, with some modifications, was used. The main variation was the addition of 0.5% glucose to the medium, which improved the protein yield due to catabolite repression, as already observed for other human amino acid transporters [30,31]. In brief, a colony was inoculated overnight at 37 °C. On the next day, a 1:20 dilution in fresh LB medium supplemented with antibiotics and glucose was performed. The cell growth was monitored by measuring optical density and, during the exponential growth phase, 0.4 mM IPTG was added to induce protein synthesis at 28 °C. After 4 h, the cells were harvested, and the bacterial pellet was resuspended in a specific buffer (see Section 4), sonicated, and centrifuged. The *Vv*CAT2 protein was enriched in the insoluble fraction of the induced cell lysate (Figure 1a, lane 2); the presence of *Vv*CAT2 in this fraction was demonstrated by the immuno-staining with the anti-His antibody (Figure 1, lane 2).

For *Vv*CAT2 purification, the Ni^2+^ chelating affinity chromatography procedure was adopted by exploiting the 6His tag added at the C-terminus of the protein. The wash fractions using the solubilization buffer did not contain the *Vv*CAT2 (Figure 1, lanes 3 and 4), indicating that the protein was bound to the resin. Then, the protein was eluted with imidazole as a single protein band (Figure 1, lane 5). The on-column refolding strategy, previously used for the *Sl*CAT2 transporter, was modified for optimal purification. In the case of *Vv*CAT2, Cholesteryl HemiSuccinate (CHS) was added during the solubilization of *Vv*CAT2 to improve both the solubility and stability of the protein, as previously done for other human transporters [30,32]. CHS is a more soluble cholesterol derivative often used for assisting with the solubilization of membrane transporters, since this compound stabilizes the interaction of the protein with the detergents by interacting with the hydrophobic moieties of membrane proteins [30]. The protein yield was 0.16 mg/L of cell culture. This procedure proved useful for obtaining the purified protein in a functionally active form [33].

### 2.2. Functional Characterization and Regulation

**Figure 2:**
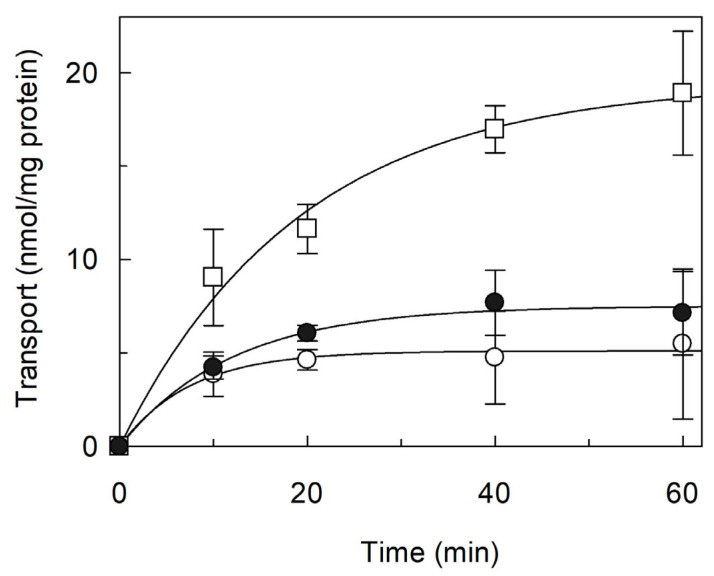
Effect of Cholesteryl HemiSuccinate (CHS) on transport activity of *Vv*CAT2. *Vv*CAT2 was purified and reconstituted in proteoliposomes, as described in Section 4. Transport was measured by adding 100 µM [^3^H]arginine to proteoliposomes containing 15 mM ATP. Proteoliposomes were prepared in the absence (○) or in the presence of 0.5 mg (●) or 1 mg (□) CHS, which is a more soluble analogue of cholesterol. Data were plotted by first-order rate equation. Results are means ± SD from three different experiments (*n* = 3).

Transport of [^3^H]arginine was investigated in proteoliposomes reconstituted with *Vv*CAT2, as previously done for *Sl*CAT2 [22]. Indeed, based on the 71% identity shared between the two proteins (Supplementary Figure S2), we expected similar transport features. To achieve the best conditions for reconstitution into proteoliposomes, the detergent/phospholipid ratio was optimized, as previously done for *Sl*CAT2 [22]. The best conditions in terms of optimal transport activity were obtained with a 0.7 detergent/lipid (*w*/*w*) ratio and a 0.001 protein/phospholipid (*w*/*w*) ratio. Then, the time dependence of [^3^H]arginine uptake into the proteoliposomes was studied. The data obtained could be fitted by a first-order rate equation, in agreement with a protein-mediated process (Figure 2). The proteoliposome system gives the possibility to test the effect of possible regulators, such as osmotic pressure, nucleotides and lipids. The content of cholesterol in *Arabidopsis thaliana* is known to be about 6% of the sterol content, which can also be higher in other plants [34,35]. The activity of many eukaryotic transporters (among which are amino acid transporters) is modulated by cholesterol [36,37,38,39]. Thus, CHS, used for protein solubilization, was also included in the proteoliposomal membrane harboring *Vv*CAT2. The transport activity of *Vv*CAT2 was measured in the presence of two different CHS concentrations (Figure 2). [^3^H]arginine uptake was strongly stimulated by CHS, suggesting that interaction with the cholesterol analogue facilitates the conformational changes required for the transport function.

**Figure 3:**
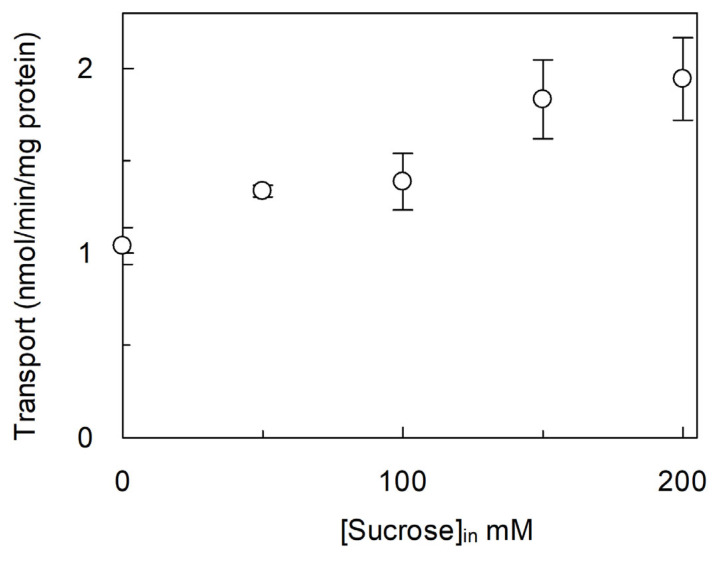
Effect of osmotic pressure on transport activity of *Vv*CAT2. *Vv*CAT2 was purified and reconstituted in proteoliposomes, as described in Section 4. Transport was measured in 20 min by adding 100 µM [^3^H]arginine to proteoliposomes containing 15 mM ATP and the indicated concentrations of sucrose. Results are means ± SD from three different experiments (*n* = 3).

It is known that sucrose accumulates in the vacuole as a temporary storage pool [40]. Therefore, the possible influence of sucrose on the [^3^H]arginine uptake mediated by *Vv*CAT2 was evaluated by studying the dependence of the transport rate on the sucrose concentration in the intraliposomal compartment (Figure 3). The [^3^H]arginine transport rate, measured as previously described, was doubled in the presence of 200 mM intraliposomal sucrose, indicating that the transporter is sensitive to an osmotic imbalance.

**Figure 4:**
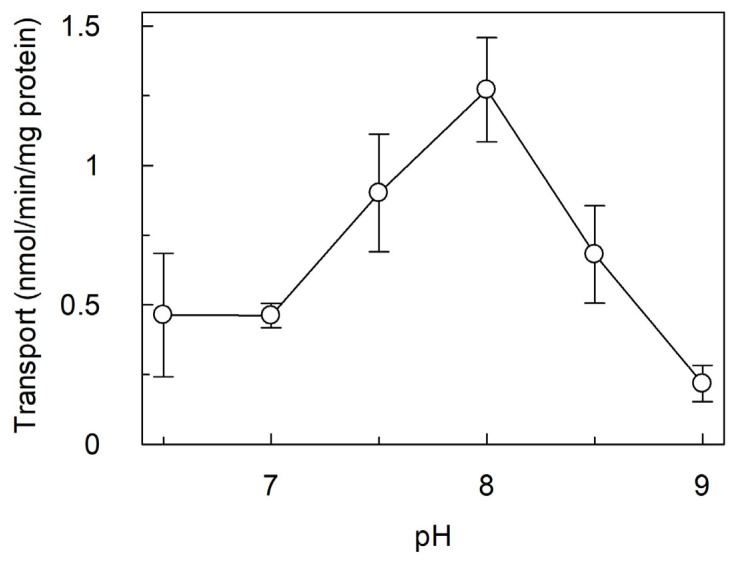
Effect of pH on transport activity of *Vv*CAT2. *Vv*CAT2 was purified and reconstituted in proteoliposomes, as described in Section 4. Transport was measured in 20 min by adding 100 µM [^3^H]arginine to proteoliposomes containing 15 mM ATP. The pH conditions were varied and kept equal in both internal and external proteoliposome compartments. Results are means ± SD from three different experiments (*n* = 3).

The dependence of the transport activity of *Vv*CAT2 on the pH was evaluated in a range from pH 6.5 to pH 9.0. As shown in Figure 4, the pH dependence of [^3^H]arginine uptake showed an optimum of activity at pH 8.0, similar to *Sl*CAT2.

**Figure 5:**
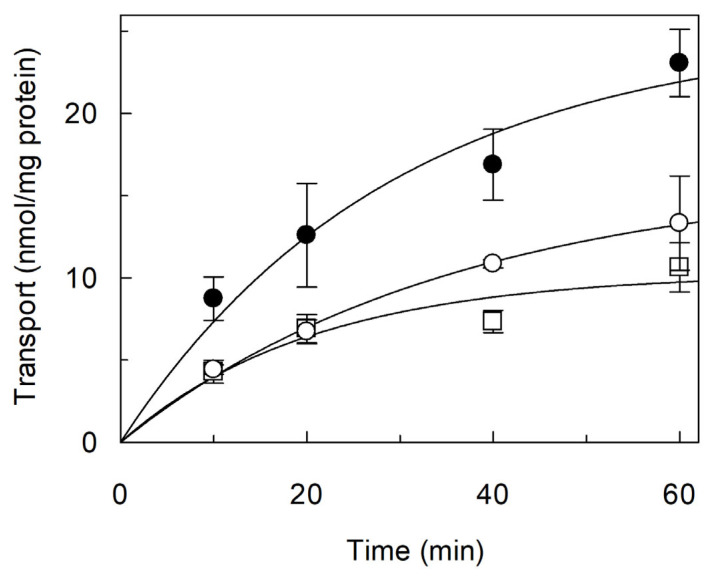
Effect of ATP on transport activity of *Vv*CAT2. *Vv*CAT2 was purified and reconstituted in proteoliposomes, as described in Section 4. Transport was started by adding 100 μM [^3^H]arginine to proteoliposomes reconstituted in absence (○) or presence (●) of intraliposomal 15 mM ATP. As control, according to preparations of commercially available ATP, ATP was replaced by 30 mM NaCl (□). Data were plotted by first-order rate equation. Results are means ± SD from three different experiments (*n* = 3).

Considering that some vacuolar transporters are allosterically activated by intravacuolar ATP [41], the effect of ATP on the [^3^H]arginine transport mediated by the reconstituted *Vv*CAT2 was investigated. To this aim, the transport rate was measured in the presence of ATP in the internal proteoliposome compartment, which may correspond to the intravacuolar compartment. The basal, time-dependent transport of [^3^H]arginine, i.e., CAT2 mediated transport in the absence of ATP (Figure 5; open circle), was strongly stimulated by internal ATP (Figure 5; black circle). The stimulation was about 96% when measured on the transport rate calculated as the product of k (the first-order rate constant) and the transport at an infinite time, extrapolated from the time course, and giving a transport rate of 0.46 or 0.9 nmol/min × mg of protein in absence or presence of internal ATP, respectively. To ascertain that the observed activation was specifically due to ATP and not to Na^+^ contained, Na^+^ in the form of NaCl was included in the place of ATP in the proteoliposomes; Na⁺ had no stimulatory effects, confirming the specificity of ATP (Figure 5; open square). As a further control, ATP was also included in the liposomes without any incorporated protein: in this case, no stimulation of radioactive substrate entry was detected, again in favor of a transporter-mediated and regulated process.

**Figure 6:**
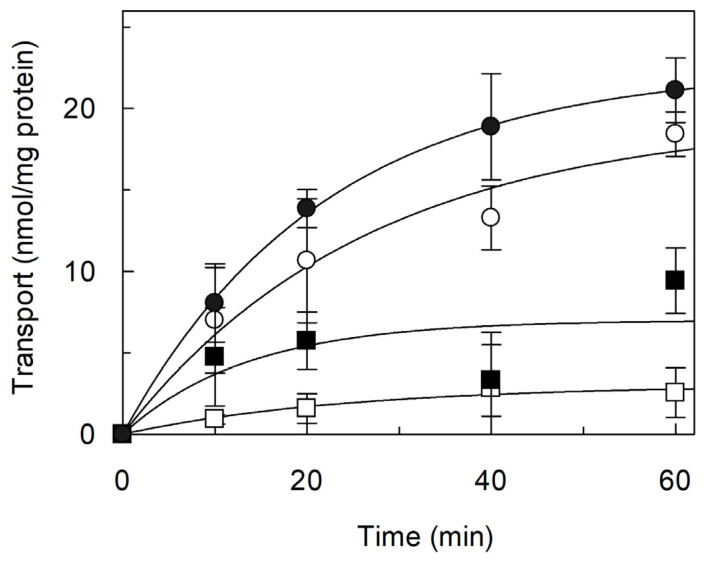
Transport of other substrates mediated by reconstituted *Vv*CAT2. *Vv*CAT2 was purified and reconstituted in proteoliposomes as described in Section 4. Transport was started by adding 100 μM of [^3^H]arginine (●) or [^3^H]ornithine (○), or [^3^H]glutamine (□) or [^3^H]histidine (■) to proteoliposomes containing 15 mM ATP. Data were plotted by first-order rate equation. Results are means ± SD from three different experiments (*n* = 3).

The transport of other nitrogen-rich amino acids, such as ornithine, glutamine and histidine, was also investigated (Figure 6). The transport of [^3^H]ornithine was slightly lower than that of [^3^H]arginine, whereas the transport of [^3^H]histidine was much lower than that of [^3^H]arginine. In contrast, the transport of [^3^H]glutamine was almost negligible.

**Figure 7:**
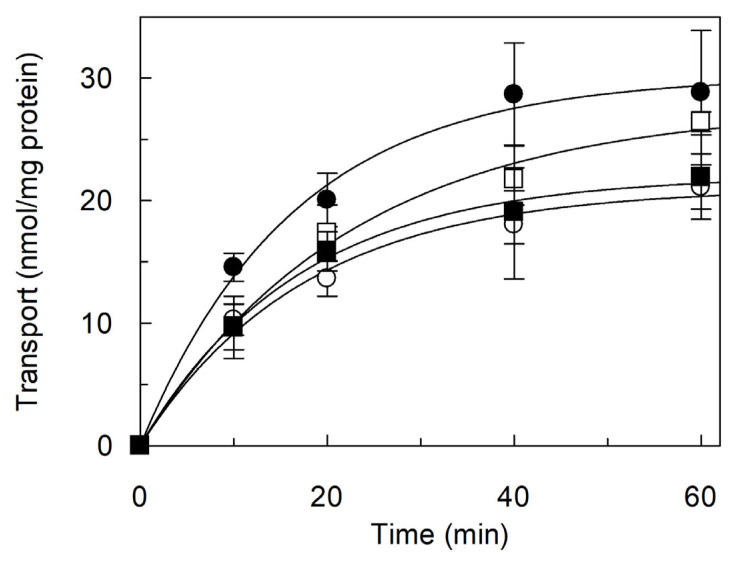
Effect of counter substrates on transport activity of *Vv*CAT2. *Vv*CAT2 was purified and reconstituted in proteoliposomes as described in Section 4. Transport was started by adding 100 μM of [^3^H]arginine to proteoliposomes containing 15 mM ATP in absence of internal substrate (○), or in presence of 10 mM internal arginine (●) or ornithine (□). As control, 10 mM internal sucrose was introduced in proteoliposomes in place of substrates (■). Data were plotted by first-order rate equation. Results are means ± SD from three different experiments (*n* = 3).

The arginine transport mode was investigated to distinguish between uniport and antiport modes. To this aim, the uptake of [^3^H]arginine was measured in the absence of any internal substrate, which is a condition corresponding to a uniport mode. In other samples, non-radioactive arginine or ornithine was added to the intraliposomal compartment at a concentration of 10 mM, establishing conditions for measuring antiport transport. The transport activities measured under the different conditions were similar (Figure 7); it has to be stressed that 10 mM sucrose had no osmotic effect, as shown in Figure 3. The presence of arginine or ornithine in the intraliposomal compartment had only a slight trans-stimulation effect on [^3^H]arginine transport, overall indicating a uniport mode of transport. Indeed, in the case of antiport transporters, the stimulation of uptake by the internal/external substrate gradient is expected to be at least four times higher, as was recently described for a serine transporter [30].

**Figure 8:**
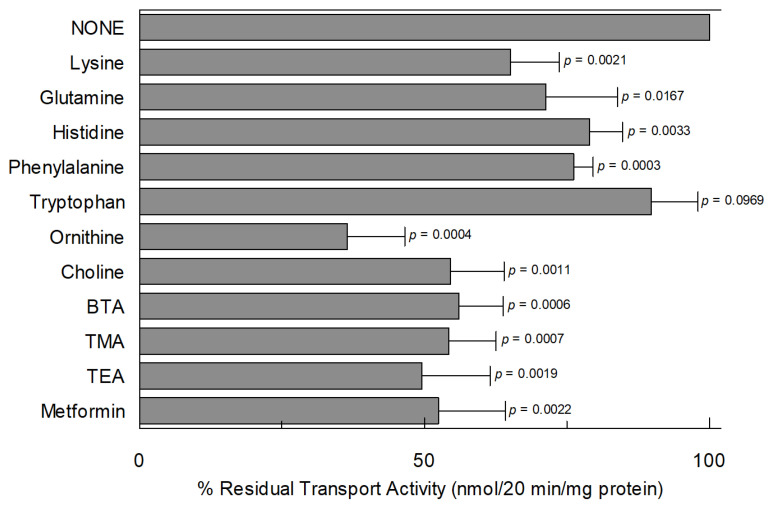
Effect of inhibitors on transport activity of *Vv*CAT2. *Vv*CAT2 was purified and reconstituted in proteoliposomes as described in Section 4. Transport was measured in 20 min by adding 100 μM [^3^H]arginine along with 10 mM of indicated molecules to proteoliposomes containing 10 mM arginine and 15 mM ATP. Percentage of residual transport activity (nmoles/20 min/mg proteins) with respect to control (absence of inhibitor) is reported. Results are means ± SD from three different experiments (*n* = 3). Significantly different at *p* < 0.01, as calculated from Student’s *t* test analyses. Exact *p* values are reported. BTA (benzyltriethylammonium); TMA (tetramethylammonium); and TEA (tetraethylammonium).

The specificity of *Vv*CAT2 towards various amino acids and other compounds was also investigated by adding the various compounds to the proteoliposomes together with [^3^H]arginine and evaluating their inhibitory effect (Figure 8). In agreement with the ability of *Vv*CAT2 to catalyze ornithine transport, this amino acid also inhibited arginine uptake of about 75%, whereas a very low, if any, level of inhibition was observed for the other amino acids, such as histidine, glutamine and lysine, correlating with low transport activity. Low or not significant inhibition by the hydrophobic amino acids phenylalanine or tryptophan, respectively, was found. Cationic substrates, such as the prototype of organic cations, tetraethylammonium (TEA), choline, benzyl-triethyl ammonium (BTA), tetramethylammonium (TME), and the hypoglycemic cationic drug metformin, were also tested; these molecules exerted an inhibition of roughly 50% for arginine transport at the tested concentration.

**Figure 9:**
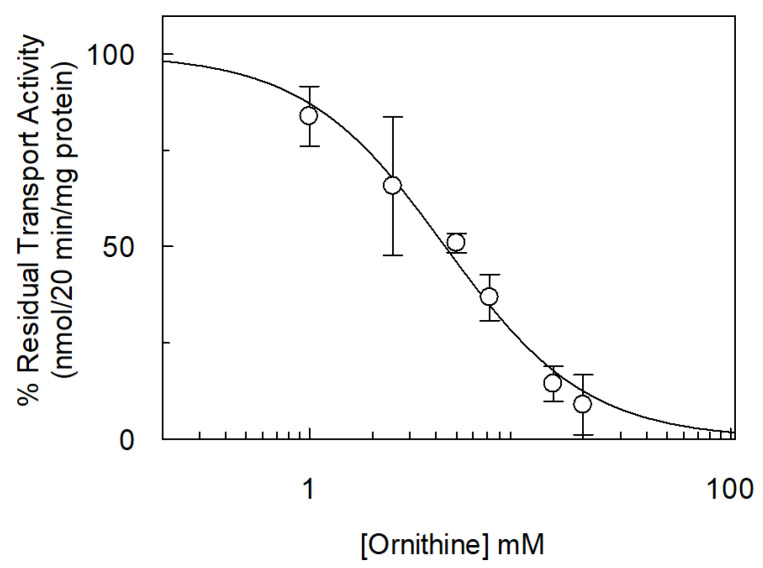
Dose–response analysis of inhibition of *Vv*CAT2 by ornithine. *Vv*CAT2 was purified and reconstituted in proteoliposomes as described in Section 4. Transport was measured in 20 min by adding 100 µM [^3^H]arginine together with indicated concentrations of ornithine to proteoliposomes containing 10 mM internal arginine and 15 mM ATP. Percentage of residual transport activity (nmoles/20 min/mg proteins) with respect to control (absence of inhibitor) is reported. Results are means ± SD from three different experiments (*n* = 3).

The ornithine inhibition, which was the strongest, was further kinetically investigated. First, a dose-response analysis was performed (Figure 9). [^3^H]arginine uptake was measured in the presence of externally added ornithine at increasing concentrations. The calculated IC_50_ value was 4.42 ± 0.98 mM.

**Figure 10:**
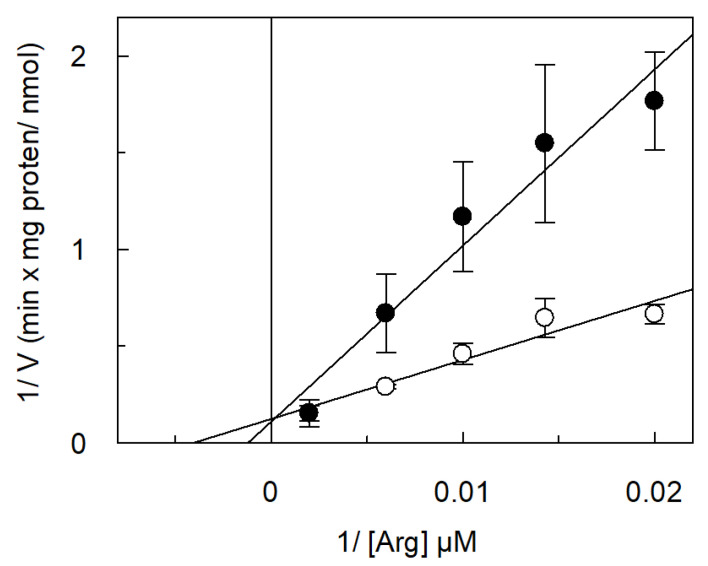
Kinetic analysis of ornithine inhibition. *Vv*CAT2 was purified and reconstituted in proteoliposomes as described in Section 4. Transport rate was started by adding [^3^H]arginine at indicated concentrations to proteoliposomes containing 10 mM internal arginine and 15 mM ATP. Transport was measured in 15 min in absence (○) or presence of (●) 5 mM ornithine. Data are analyzed, according to Lineweaver–Burk, as reciprocal transport rate versus reciprocal arginine concentration. Results are means ± SD from three different experiments (*n* = 3).

A kinetic analysis of arginine transport and inhibition kinetics in the presence of ornithine was performed (Figure 10). The calculated arginine Km was 252 ± 33.2 µM. In the same experiments, the Ki for ornithine was 2.2 ± 0.5 mM.

**Figure 11:**
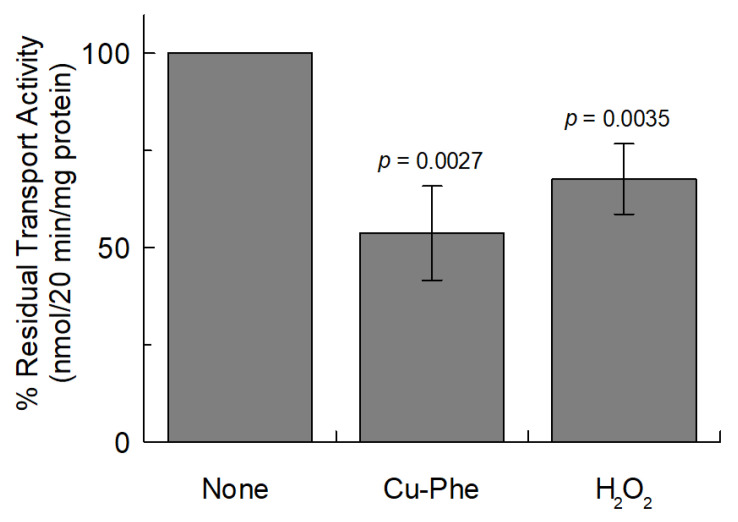
Effect of oxidant compounds on transport activity of *Vv*CAT2. *Vv*CAT2 was purified and reconstituted in proteoliposomes as described in Section 4. Transport was measured in 20 min by adding 100 µM of [^3^H]arginine, in absence (none) or presence of oxidants copper phenanthroline (Cu-Phe) or hydrogen peroxide (H_2_O_2_), to proteoliposomes containing 10 mM internal arginine and 15 mM ATP. Percentage of residual transport activity (nmoles/20 min/mg proteins) with respect to control (absence of inhibitor) is reported. Results are means ± SD from three different experiments (*n* = 3). Significantly different a *p* < 0.01, as calculated from Student’s *t* test analyses. Exact *p* values are reported.

It is well known that plant metabolism is sensitive to oxidative stress [42]. Therefore, we have tested whether oxidative conditions caused by Cu-Phenanthroline or hydrogen peroxide could play a role in the transport function of *Vv*CAT2 (Figure 11).

Indeed, treatment with both compounds at concentrations frequently used in vitro [43] significantly impaired the transport function, with more than a 40% loss of transport activity. This indicates that the reconstituted transporter responds to conditions of oxidative stress.

## 3. Discussion

In plants, the interest in the study of transporters has recently been related to the interest in plant resistance to abiotic stress and crop improvement, which is focused on human nutrition and health [44,45]. Amino acid transporters have been reported to enhance plant tolerance to abiotic stress, resistance to external bacterial invasion and various environmental stimuli, which are also caused by climate change. Therefore, the knowledge and characterization of these transporters are of special interest for viticulture, which nowadays faces various abiotic and biotic threats, such as salinity stress, drought stress and pathogen attack. This role of amino acid transport has so far been overlooked [16], also due to a lack of appropriate methodology for studying membrane transporters. In this frame, the multiple roles of the Cationic Amino acid Transporters (CATs) in *A. thaliana* and other species have been elucidated. For example, *At*CAT2 was found to be responsive to light, low temperatures and circadian rhythms, and is critical for plant growth and development, nutrient metabolism and stress resistance. *Brassica napus* CATs, *Bn*CATs, were responsive to different nutrient stresses [23]. Other roles of CATs in drought and stress tolerance have also been demonstrated [20]. Moreover, the role of CATs in the generation and scavenging of ROS has been recently described [15].

In this work, we describe a new study on a CAT2 transporter from a different species: *V. vinifera*. This study has been undertaken due to the importance of this plant species in human nutrition, with the perspective of improving the production and extraction of secondary metabolites that could be used in the prevention and treatment of human diseases. *VvCAT2* cDNA has been successfully cloned and expressed in *E. coli* Rosetta(DE3)pLysS. In this strain, the combination of the T7lac promoter within the vector and the DE3 lysogens strain results in tight control of the transcription level, preventing leaky expression; this is crucial for membrane transporter expression. The approach led to obtaining a protein amount high enough for functional characterization in proteoliposomes. This *E. coli* strain was previously used for obtaining over-expression of CAT2 from *S. licopersicum* [22]. In contrast, the same strain was not suitable for some human transporters, such as SLC7A10 [30] and SLC38A2 [31], for which other strains had to be employed. These results indicate that the conditions for heterologous expression may change for genes of different kingdoms. The proteoliposome model allows us to assay transport in the absence of interferences caused by other transporters and/or enzymes that are present in intact cells. Moreover, the predicted localization of *Vv*CAT2 in vacuoles could prevent the measurement of transport activity in intact plant cells. In favor of vacuolar localization, we have found a dependence of the transporter on the osmolality gradient, which is a typical feature of vacuoles [46]. Based on the stimulation by internal hyperosmolarity, it is plausible that the intraliposomal face of the transporter could correspond to the intravacuolar one. However, no direct evidence of a vacuolar localization has been reported so far and, hence, at this stage, we cannot definitively assess that the intraliposomal side of the protein corresponds to the intravacuolar side. Several common features of *Vv*CAT2 with respect to *Sl*CAT2 have been revealed. Among these are the specificity towards arginine and the stimulation of transport by ATP. Another interesting effect observed on the two transporters was the stimulation by the cholesterol analogue CHS. These results highlight that *Vv*CAT2 exhibits regulatory properties. However, further studies of structure/function relationships are needed to reveal the molecular basis of this regulation. The transporter is sensitive to the thiol oxidant copper phenanthroline, suggesting a possible involvement in ROS sensing. The arginine Km in *Vv*CAT2 was slightly lower than that of *Sl*CAT2. The arginine transport mediated by *Vv*CAT2 was inhibited by ornithine by a competitive mechanism, indicating that the two amino acids bind to the same protein binding site, as was also found for *Sl*CAT2. Arginine is a major signaling molecule and a key regulator of the mTORC1 pathway. Moreover, it is a precursor of both ornithine and nitric oxide (NO). In plants, besides these common roles, arginine modulates the production of secondary metabolites involved in fruit development and stress tolerance. Indeed, arginine application has been found to exert a positive effect on fruit quantity and quality, stimulating the accumulation of antioxidants such as ascorbic acid, phenol compounds and anthocyanins, as well as the production of phytohormones [47]. Therefore, we may hypothesize that *Vv*CAT2 could be involved in processes linked to improving the yield and quality of crops [47]. The slight sensitivity of the transporters to inhibition by phenylalanine, which is involved in secondary metabolite pathways, correlates with a possible role of the transporter in these pathways.

In addition to antioxidants, arginine, which is transported by *Vv*CAT2, is the precursor of polyamines that are among the best-recognized secondary arginine-derived metabolites. They act as growth regulators and, being associated with cell division, could be used to regulate fruit development and protect against abiotic stress. Thus, manipulating pathways involving arginine metabolism and transport could have fallouts on human nutrition. Indeed, many secondary metabolites with antioxidant or anti-inflammatory properties are derived from or are influenced by arginine. This, in turn, could improve the composition of plant extracts to be used as nutraceuticals/phytoceuticals. These compounds have enormous promise for the prevention and treatment of diseases like cancer, cardiovascular diseases, neurodegenerative disorders and others [48].

Plant vacuoles have been primarily recognized as a site for the accumulation of secondary metabolites [28,49,50]. In this sense, a prominent role is hypothesized for tonoplast transporters, but the overall knowledge about the transporters responsible for the movement of precursors or intermediates across the vacuolar membrane is still limited [28]. The results described here represent an important first step towards developing knowledge of a transporter, that is, the involvement of *Vv*CAT2 in arginine transport. This would suggest a key role is played by this transporter in plant nitrogen trafficking, thus representing a target for crop improvement with relevance to human nutrition and health.

## 4. Materials and Methods

### 4.1. Plant Material

Fresh leaves of *V. vinifera* were collected from the Botanical Garden of the University of Calabria. Tissues were immediately frozen in liquid nitrogen at −80 °C until processed.

### 4.2. RNA Extraction, cDNA Production and Cloning of VvCAT2

The total RNA was isolated from grape berry flowers using the RNeasy Plant Mini Kit (Qiagen, Germantown, MD, USA), following the instructions of the manufacturer. Then, 1 µg total RNA was subjected to reverse transcription using the SuperScript^®^ III First-Strand Synthesis for RT-PCR system (Invitrogen, Waltham, MA, USA). The obtained first-strand cDNA was used as the template for amplifying the full-length *VvCAT2* cDNA by PCR, using primers designed according to the conserved region of the homologous gene transcript in GenBank (accession number XM_002271294.4). The forward (*Vv*fw 5′-CTAAAGCTTCCATGGGTGTGGGTGGTGATTTTG-3′) and reverse (*Vv*rev 5′-GCGCTCGAGAGCTAAGCAGTCTGA-3′) primers contained the *Hind*III and *Xho*I restriction sites, respectively. These sites were adopted for cDNA cloning into the pET-21a(+) expression vector (Novagen, Bethesda, MD, USA). The obtained recombinant plasmid, called pET21-*VvCAT2*-6His, was sequenced by the Eurofins Genomics DNA sequencing service (https://www.eurofinsgenomics.eu, accessed on 15 May 2023).

### 4.3. Protein Production

The expression of *Vv*CAT2 was obtained thanks to the crucial tRNA supply contribution provided by the *E. coli* Rosetta(DE3)pLysS strain (Sigma Aldrich, St. Louis, MO, USA). The bacteria transformed with the pET21-*VvCAT2*-6His construct were inoculated in LB medium, supplemented with 100 µg/mL ampicillin, 34 µg/mL chloramphenicol and 0.5% glucose, which improved protein expression. Then, bacteria were grown overnight (37 °C) under rotatory shaking at 160 rpm. After 24 h, the inoculum was diluted 1:20 in fresh selective LB medium supplemented with 0.5% glucose. When the OD (600 nm wavelength) reached ~0.8, 0.4 mM isopropyl-β-d-thiogalactopyranoside (IPTG) was added to induce protein expression. Then, to maximize protein production, the temperature of the bacterial culture was lowered to 28 °C for 4 h. After this step, the cells were harvested by centrifuging at 6000× *g* (10 min, 4 °C). The pellets were resuspended in 20 mM Hepes-Tris, pH 7.5, 300 mM NaCl. Cells were then subjected to sonication at 4 °C (5 min, 30% power 1 s sonication, 1 s intermission) with a Branson SFX550 sonifier (VWR International s.r.l., Milano, Italy). The insoluble fraction was separated from the soluble one by centrifugation (15,000× *g* for 7 min at 4 °C) and subjected to 12% SDS-PAGE and Western Blot.

### 4.4. Protein Purification

The insoluble fraction from 16 mL of IPTG-induced cell lysate was washed twice with 100 mM Tris HCl, pH 8.0, then centrifuged at 20,000× *g* (10 min). The pellet was re-suspended in 1600 µL 8 M urea, 80 µL 500 mM DTE, 216 µL 10% sarkosyl/2% CHEMS and 1 mL of buffer A, containing 0.1% sarkosyl/0.02% CHEMS, 200 mM NaCl, 10% glycerol and 20 mM Tris HCl, pH 8.0, and then centrifuged for 15 min at 20,000× *g*. Three mL of the supernatant were applied onto a column filled with His-select Ni-Chelating affinity gel (Sigma Aldrich, St. Louis, MO, USA) (0.5 cm diameter, 3 cm height) preconditioned with 8 mL of buffer A. The column was eluted with 5 mL of a wash buffer containing 0.1% DDM/0.02% CHEMS, 5 mM DTE, 200 mM NaCl, 10% glycerol and 20 mM Tris HCl, pH 8.0, then 20 mL of the same buffer with 10 mM imidazole, then 8 mL of the same buffer with 50 mM imidazole. Sixteen fractions of 500 µL were collected. The eighth to the eleventh fractions were collected for recovering the purified protein.

### 4.5. Reconstitution of the SlCAT2 Transporter into Liposomes

Liposomes were obtained by suspending 100 mg of L-α-Phosphatidylcholine in 1 mL of water, sonicating the suspension for 2 min at 0 °C (1 s sonication, 1 s intermission), and then centrifuging the sonicated liposomes at 14,000× *g* for 3 min at room temperature to remove undissolved aggregates. The purified *Vv*CAT2 was reconstituted using a method based on the detergent removal from mixed micelles composed of protein, detergent and liposomes to obtain proteoliposomes. The removal procedure was performed by incubating the mixture with Amberlite XAD-4 (Sigma Aldrich) using a batch-wise procedure [51]. The composition of the initial reconstitution mixture was 300 μL purified protein (12 µg in 0.1% DDM) or 300 μL of the purification buffer (for performing controls), 5 μL 0.3 M EDTA, 280 μL of a solution composed of 120 μL 10% egg yolk phospholipids in the form of sonicated liposomes, obtained as previously described, and 160 μL of 5% Triton-X 100/0.75 mg CHEMS, 41 μL 273 mM ATP and 10 mM Tris-Hepes, pH 7.5, in a final volume of 700 μL. The mixture was vortexed for 4 min and then incubated under rotatory stirring (1200 rpm) with 0.5 g Amberlite XAD-4 at 23 °C for 40 min.

### 4.6. Transport Measurements

After the reconstitution procedure, 550 μL of the obtained proteoliposomes were applied on a Sephadex G-75 column (Sigma Aldrich, St. Louis, MO, USA) (0.7 cm diameter × 15 cm height) pre-equilibrated with 10 mM Tris/Hepes pH 7.5. From these columns, 550 μL of proteoliposomes were collected and divided into aliquots (samples) of 100 μL. Transport was started by 100 μM [^3^H]arginine addition (or other radioactive substrates, as indicated in the figure legends) to the proteoliposome samples. In control samples (liposomes without incorporated protein), transport was measured by the same procedure. The assay temperature was 25 °C. Transport was stopped by passing 100 μL of each sample through a Sephadex G-75 column (0.6 cm diameter × 8 cm height) to remove the external (not taken up) radioactivity. Samples were eluted with 1 mL of 50 mM NaCl and collected in 2.8 mL of scintillation mixture for intraliposomal radioactivity counting. The experimental values were corrected by subtracting the controls (liposomes without incorporated protein). The final transport activity was expressed as nmol × min^−1^ × mg protein^−1^. The time course data were interpolated by a first-order rate equation from which transport rates were derived as the product of k (the first-order rate constant) and the transport at the equilibrium. Kinetics were performed by measuring transport at 15 min, i.e., in the linear range of time courses. Experimental data were plotted using first-order rate, dose–response or Lineweaver–Burk equations by the Grafit (version 5.0.13) software.

### 4.7. Other Methods

CHS was solubilized as previously described [37]. Electrophoresis was conducted as previously described [51].

## Data Availability

The original contributions presented in this study are included in the article/Supplementary Materials. Further inquiries can be directed to the corresponding author(s).

## Supplementary Materials

The supporting information can be downloaded at https://www.mdpi.com/article/10.3390/ijms26136259/s1.

## Abbreviations

APC	Amino acid-Polyamine/Choline
ATF	Amino acid Transporter superFamily
BTA	Benzyltriethylammonium
CAT	Cation Amino acid Transporter
CHS	Cholesteryl HemiSuccinate
Cu-Phe	Copper Phenanthroline
DDM	n-Dodecyl-β-D-Maltoside
DTE	Dithioerythritol
EDTA	Ethylenediaminetetraacetic Acid
IPTG	Isopropil-β-D-tiogalattopiranoside
TEA	Tetraethylammonium
TMA	Tetramethylammonium
