# Supplementary material for: Cloning, Expression and Functional Characterization of V. vinifera CAT2 Arginine Transporter
Source: Int J Mol Sci. 2025 Jun 28;26(13):6259. doi: 10.3390/ijms26136259 (PMC12250071; doi:10.3390/ijms26136259)
Supplement: Supplementary file 1 [file ijms-26-06259-s001.zip › ijms-3683207-supplementary.pdf]

ATGGGTGTGGGTGGTGATTTTGATTCACAGAAGAGGGGTTCAAGGGGATTCAAAGGATTGATGAGGAGAAAGCTGGTTGATTCTGCCAGAAAG
GAGGTGGATGGTGGTCATAAATTGGCCAAGGAATTATCTGTGACCCATCTTATTGCTATTGGAGTTGGGTCAACAATTGGGGCTGGTGTTTATATC
CTAGTTGGAACTGTGGCAAGGGAGCATTCAGGGCCTGCACTCACAATTTCATTTCTAATAGCTGGAATAGCAGCTGCACTTTCAGCCTTTTGCTAT
GCAGAGCTTTCTAGTCGTTGCCCATCTGCCGGGAGTGCCTACCATTATTCCTACATATGTGTTGGTGAAAGTGTTGCTTGGTTGATTGGTTGGGCAT
TGATTCTGGAATATACAGTTGGTGGTTCAGCAGTTGCACGCGGCATATCCCCAAATCTGGCCTTGCTGTTTGGAGGTGGGGATCTACCTGCTTTTA
TAGCCCGTCAGTATATTCCAGCGCTTGATATTGTGGTTGACCCATGTGCAGCAATTTTAGTTTTTATTGTCACTGGGCTCTTGTGTGTGGGAATCAA
GGAGAGTACAGTTGCACAAGCCATTGTCACAACGGGAAATGTATGTGCCATGATGTTTGTCGTAATAGCTGGTGGATATCTTGGTTTCAAGACTG
GATGGGCTGGATATGAACTTCCTACTGGGTATTTTCCTTTTGGAGTAGATGGTATGCTGGCTGGGTCTGCAACAGTCTTCTTTGCATACATTGGTTT
TGATTCTGTTGCCAGCACAGCTGAGGAGGTGAAGAATCCCCAGCGAGATTTGCCACTGGGTATTGGTGCTGCACTGTCGATATCCTGCATACTAT
ATATGTTGGTCTCTGTTGTCATTGTTGGTCTGGTGCCCTATTATTCAATGGATCCTGACACCCCCATCTCCACTGCATTTGCTAGCCATGGGATGAA
ATGGGCAGCGTACATAATAACTATTGGAGCTGTTACTGCTCTCTGCTCAACATTGATGGGTTCACTCCTCCCTCAGCCGCGAATCCTGATGGCAAT
GGCTAGAGATGGATTGCTGCCGTCATTTTTTTCAGATCTCAACAGGCGCACACAAGTTCCTGTCAAGGCCACAATAGTAACTGGTATTGGGGCTG
CAACTCTGGCTTTCTTTATGGATGTTTCACAATTGGCAGGAATGGTCAGTGTGGGCACACTTCTTGCCTTCACCATGGTAGCATGTTCTGTGTTAAT
ACTCCGATATGTTCCACCCGATGAAGTGCCACTCACACCATCACATCAGGAATCAATTGATACAGTTTCATTACAACATAGCAGTAGCAACCAGA
AGATTGATGAGGAAAATTCCAAAGTTTGTGCTGGCCCCTCTAATGAGATTACTCGACCTTTAATtGCTAAAGAGGGAGCATCAGCTGATCCTGTTG
TTGTAAAAAATGTAGTTCTAGACAGATACgTAATAGATGAAAACATGAGGCGAAAAATTGCTGGCTGGACCATAATGCTGACATGTGTAGGAGT
GGTTCTCCTTACATCTGCTGCTTCGTATGTGAACCTTCCCAGCTTTCCTCGTTACATGTTTTGTGGAATCGGTGGTGCTTTACTTTTATCTGGTCTGA
TAGTGCTCACCTGTATAGATCAAGATGATGCAAGGCACAACTTTGGGCATTCAGGAGGTTTCATTTGCCCATTCGTCCCACTCCTACCTATTGCCT
GCATTCTCATCAACGTCTACTTATTGGTGAATCTTGGATCTGCTACCTGGACCCGGGTTTCTATATGGTTGGGGATAGGAGTGCTCGTTTATGGATT
CTATGGGCGAAGACAtAGCTCACTGCAGAATGCAGTTTATGTACCAGCAGCTGATGTGGATGAAATCTATGGAAGCTCTTCAGACTGCTTAGCTT
AG

Figure S1. CDS sequence of *VvCAT2*

```
SlCAT2    MGFVGDGSCSSSGGDKGGCFIGGMKSLVRRKQVDSANSKSSSTSGSSHQLAKALTIPHLI
VvCAT2    MGVGGDFDSQKRGS-------RGFKGLMRRKLVDSARKEVDG----GHKLAKELSVTHLI
          **. ** .... *.        *:*.*:*** ****..: ..    .*:*** *::.***

SlCAT2    TIGVGSTIGAGVYILVGTVAREHSGPALTISFLIAGIAAALSAFCYAELASRCPSAGSAY
VvCAT2    AIGVGSTIGAGVYILVGTVAREHSGPALTISFLIAGIAAALSAFCYAELSSRCPSAGSAY
          :************************************************:**********

SlCAT2    HYSYICVGEGVAWLIGWALVLEYTIGRSAVARGISPNLAMLFGSPDSLPSFLARHTIPGL
VvCAT2    HYSYICVGESVAWLIGWALILEYTVGGSAVARGISPNLALLFGGGD-LPAFIARQYIPAL
          *********.*********:****:* ************:***. * **:*:**: **.*

SlCAT2    NITVDPCAAILVFLVTGLLCVGIKESTVVQGFVTSVNVCVMAFVIIAGGYLGYKAGWPGY
VvCAT2    DIVVDPCAAILVFIVTGLLCVGIKESTVAQAIVTTGNVCAMMFVVIAGGYLGFKTGWAGY
          :*.**********:**************.*.:**: ***.* **:*******:*:**.**

SlCAT2    ELPVGYFPYGVDGMLAGASTVFFAYIGFDSVASTAEEVKNPQRDLPMGIGFALSICCSLY
VvCAT2    ELPTGYFPFGVDGMLAGSATVFFAYIGFDSVASTAEEVKNPQRDLPLGIGAALSISCILY
          ***.****:********::***************************:*** ****.* **

SlCAT2    MLVSAVIVGLVPYYAMDPDTPISSAFASHGINWAAYIITIGACTSLCSTLMGSIMPQPRI
VvCAT2    MLVSVVIVGLVPYYSMDPDTPISTAFASHGMKWAAYIITIGAVTALCSTLMGSLLPQPRI
          ****.*********:********:******::********** *:********::*****

SlCAT2    LMAMARDGLLPSFFSDVNKRTQVPIKGTIATGLLSGTLAFFMNVEQLSGMVSVGTLLAFT
VvCAT2    LMAMARDGLLPSFFSDLNRRTQVPVKATIVTGIGAATLAFFMDVSQLAGMVSVGTLLAFT
          ****************:*:*****:*.**.**: :.******:*.**:************

SlCAT2    MVAISVLILRYVPPDEVPVPSSYQEAIDSVRLRRSSCSSSSDMDVEKTKIPAVTSGDST-
VvCAT2    MVACSVLILRYVPPDEVPLTPSHQESIDTVSLQHS--SSNQKIDEENSKVCAGPSNEITR
          *** **************:..*:**:**:* *::*  **...:* *::*: * .*.: * 

SlCAT2    PLLG-EISVGHPLAEKAAAKLSYLVSQ--RRKVAGCTILFICIGVCIVTSAASIVNLSNP
VvCAT2    PLIAKEGASADPVVVKNVVLDRYVIDENMRRKIAGWTIMLTCVGVVLLTSAASYVNLPSF
          **:. * : ..*:. * ..   *::.:  ***:** **:: *:** ::***** ***.. 

SlCAT2    ARYALSGIGGLLLISGLIILTCIDQDDARHSFGHTGGFTCPFVPLLPIACILINVYLLIN
VvCAT2    PRYMFCGIGGALLLSGLIVLTCIDQDDARHNFGHSGGFICPFVPLLPIACILINVYLLVN
          .** :.**** **:****:***********.***:*** *******************:*
```

```
SlCAT2          LGGETWARVSIWLVIGTCIYALYGRTHSSLKTAVYVPSTHVDEIYETSAISLAC
VvCAT2          LGSATWTRVSIWLGIGVLVYGFYGRRHSSLQNAVYVPAADVDEIYGSSSDCLA-
                **. **:****** **. :*.:*** ****:.*****::.***** :*: .**
```

Figure S2. Alignment of the *S. lycopersicum* (*Sl*) and *V. vinifera* (*Vv*) amino acid sequences
